# Decreased Plasma Levels of Kynurenine and Kynurenic Acid in Previously Treated and First-Episode Antipsychotic-Naive Schizophrenia Patients

**DOI:** 10.3390/cells12242814

**Published:** 2023-12-11

**Authors:** Miloš Marković, Nataša Petronijević, Milena Stašević, Ivana Stašević Karličić, Milica Velimirović, Tihomir Stojković, Slavica Ristić, Mina Stojković, Nataša Milić, Tatjana Nikolić

**Affiliations:** 1 Clinic for Mental Disorders “Dr Laza Lazarević”, 11000 Belgrade, Serbia; milena.stasevic@yahoo.com (M.S.); ivana.stasevic.karlicic@med.pr.ac.rs (I.S.K.); 2 Faculty of Medicine, University of Belgrade, 11000 Belgrade, Serbia; natasa.petronijevic@med.bg.ac.rs (N.P.); milica.velimirovic@med.bg.ac.rs (M.V.); tihomir.stojkovic@med.bg.ac.rs (T.S.); 3 Institute of Medical and Clinical Biochemistry, Faculty of Medicine, University of Belgrade, 11000 Belgrade, Serbia; slavicaristic8@gmail.com; 4 Faculty of Medicine, University of Priština—Kosovska Mitrovica, 38220 Kosovska Mitrovica, Serbia; 5 Clinic for Neurology, University Clinical Centre of Niš, 18000 Niš, Serbia; minastojkovic@hotmail.com; 6 Institute for Medical Statistics and Informatics, Faculty of Medicine, University of Belgrade, 11000 Belgrade, Serbia; natasa.milic@med.bg.ac.rs; 7 Department for Internal Medicine, Mayo Clinic, Rochester, MN 55905, USA

**Keywords:** schizophrenia, first-episode, blood, plasma, tryptophan, kynurenine, kynurenic acid

## Abstract

Tryptophan (TRP) catabolites exert neuroactive effects, with the plethora of evidence suggesting that kynurenic acid (KYNA), a catabolite of the kynurenine pathway (KP), acts as the regulator of glutamate and acetylcholine in the brain, contributing to the schizophrenia pathophysiology. Newer evidence regarding measures of KP metabolites in the blood of schizophrenia patients and from the central nervous system suggest that blood levels of these metabolites by no means could reflect pathological changes of TRP degradation in the brain. The aim of this study was to investigate plasma concentrations of TRP, kynurenine (KYN) and KYNA at the acute phase and remission of schizophrenia in a prospective, case-control study of highly selected and matched schizophrenia patients and healthy individuals. Our study revealed significantly decreased KYN and KYNA in schizophrenia patients (*p* < 0.001), irrespective of illness state, type of antipsychotic treatment, number of episodes or illness duration and no differences in the KYN/TRP ratio between schizophrenia patients and healthy individuals. These findings could be interpreted as indices that kynurenine pathway might not be dysregulated in the periphery and that other factors contribute to observed disturbances in concentrations, but as our study had certain limitations, we cannot draw definite conclusions. Further studies, especially those exploring other body compartments that participate in kynurenine pathway, are needed.

## 1. Introduction

It was decades ago that research data revealed that tryptophan (TRP) catabolites exert neuroactive effects [1,2] with the plethora of evidence suggesting that kynurenic acid (KYNA), a catabolite of the kynurenine pathway (KP), functions as the regulator of glutamate and acetylcholine in the brain [3,4]. At elevated concentrations, KYNA competitively inhibits all ionotropic glutamate receptors [5] while in far lower concentrations competitively inhibits the glycine co-agonist site of the N-methyl-D-aspartate (NMDA) receptor [6] and as such acts as the only endogenous NMDA receptor antagonist with the potential to lead to the weakening of circuits in layer III of dorsolateral prefrontal cortex and has a putative role in schizophrenia (SZ) pathophysiology [7].

A vast majority (95%) of essential amino acid tryptophan (TRP) in the body is degraded via the complex kynurenine pathway, predominantly in the liver, while only a small proportion is converted to serotonin [8,9]. TRP can easily cross the blood-brain barrier and can be centrally engaged either in the kynurenine or serotonin pathway. Degradation of TRP (for a detailed overview of KP see [10]) by its oxidation to N-formyl kynurenine is dominantly mediated by one of three enzymes—indolamine 2,3-dioxigenase 1 (IDO1), indolamine 2,3-dioxigenase 2 (IDO2) or tryptophan 2,3-dioxigenase (TDO2). Deformylation of N-formyl kynurenine produces L-kynurenine (KYN), a central metabolite of the kynurenine pathway, both in the periphery and in the brain [10]. Under physiological conditions, activities of the mentioned enzymes in the brain are low compared to peripheral organs [11]. Juxtaposing brain to blood kynurenines shows that the brain KP is not autonomous and is under strong influence from the periphery. Besides the influx of TRP from peripheral blood to the brain, KYN and 3-hydroxykynurenine (3-HK) can also cross the blood-brain barrier unlike KYNA, which under physiological conditions does not cross the blood-brain barrier due to its polar nature and is formed locally within the brain [12,13]. Brain kynurenine metabolism and KYNA formation are mainly influenced by the entry of KYN from the blood [14]. Kynurenines are synthesized in various tissues, with notable production occurring in the liver, cells of the immune system and the brain, and the conversion of TRP to KYN is catalyzed by indoleamine 2,3-dioxygenase or tryptophan dioxygenase [15]. These enzymes are activated by different factors, like stress hormone cortisol, proinflammatory cytokines and growth factors in the periphery and the brain [16,17,18]. Elevated concentrations of proinflammatory cytokines were found in the blood of patients with chronic and first-episode schizophrenia but also in persons with an increased risk of psychosis [19]. The most replicated findings are elevated concentrations of cytokines known for their above-mentioned effect on the kynurenine pathway, i.e., IL-6, TNF-α and IL-1β across the various stages of the disease [20], while the results for two important proinflammatory cytokines, IL-8 and IFN-γ, were not consistent [20,21]. Transport through the blood-brain barrier of KP metabolites and inflammatory factors both increase the central availability of KP metabolites and additionally promote central activation of KP. These events in both the periphery and the central nervous system establish a link between neuroinflammatory mechanisms and abnormal metabolism along the kynurenine pathway, contributing to brain pathology. [22]. Centrally, KYN is metabolized either in astrocytes or in microglia, which makes separate branches of this metabolic pathway. The first branch represents irreversible transamination of KYN by the effect of kynurenine aminotransferase I, II, III and IV (KAT I-IV) in astrocytes, converting KYN to KYNA. The second branch, dominantly active in microglia, is mediated by the effects of kynurenine-monooxygenase (KMO) forming 3-HK, which is further metabolized by KAT to xanthurenic acid (XA). Besides that, in microglia, kynureninase may convert 3-HK to another metabolite, 3-hydroxyanthranilic acid. KYN is also converted in microglia by the effects of kynureninase in anthranilic acid (AA), which in the subsequent enzymatic cascade forms either quinolinic acid (QA), which is later converted into nicotinamide-adenine mononucleotide, or picolinic acid (PA) [10]. Research in the field of KP of TRP catabolism in schizophrenia does not have a short history. Studies exploring kynurenine pathway metabolites in brain and cerebrospinal fluid were very consistent and unambiguously found elevated concentrations of these metabolites in schizophrenia patients [23,24,25,26,27]. There is a significant inconsistency in results from studies that measured concentrations of kynurenic acid and other KP metabolites in the blood of schizophrenia patients [28,29,30]. Accumulating evidence regarding measures of KP metabolites in the blood of schizophrenia patients coupled with findings from the central nervous system (CNS) leads to the conclusion that blood levels of these metabolites by no means could reflect pathological changes of TRP degradation in the brain [28,29,30]. Important findings from a TRP challenge study that explored the peripheral effects of orally administered TRP show that plasma levels of KYN, KYNA and 5-hydroxyindoleacetic acid (5-HIAA) did not differ significantly between healthy individuals and clinically stable schizophrenia patients, with the conclusion that this could indicate that plasma levels of KYN and KYNA cannot be reliably used as biomarkers of schizophrenia and neither could reflect changes of CNS kynurenine and KYNA [31].

The aim of this study was to investigate differences in plasma concentrations of TRP, KYN and KYNA in patients with schizophrenia and healthy individuals and changes of these concentrations at different timepoints of the disorder and to investigate potential correlations among these concentrations and clinical characteristics as well as antipsychotic treatment. The study was conducted in a fairly big study sample of highly selected and matched schizophrenia patients and healthy individuals so that major confounders whose presence is known to affect the kynurenine pathway are excluded or controlled.

## 2. Materials and Methods

### 2.1. Participants

A total of 141 subjects were recruited in the study who were either male or female, 18 to 65 years old. The study was conducted in the Clinic for Mental Disorders “Dr Laza Lazarevic”, Clinic for Psychiatry, University Clinical Centre of Serbia and Institute for Clinical and Medical Biochemistry, School of Medicine, University of Belgrade. The study was carried out in accordance with the Code of Ethics of the World Medical Association, i.e., the Declaration of Helsinki. The study protocol was approved by the Ethics Committee of the Clinic for Mental Disorders “Dr Laza Lazarevic” (no: 8371; 28 September 2020) and the Ethics Committee of the University Clinical Center of Serbia (no: 623/9; 18 September 2020). All the participants gave their written informed consent, and their anonymity was maintained. Participants were recruited in the period from 19 March 2021 to 27 January 2022. Participants were divided into two groups—a group of schizophrenia patients and healthy controls. Schizophrenia patients met the ICD-10 criteria for the diagnosis of schizophrenia [32], and the information to support the diagnosis was collected within the clinical interview and supplemented with information from family/caregivers and previous medical documentation. Participants with schizophrenia were either in the acute relapse phase of the disorder confirmed by a Clinical Global Impression Scale score ≥4 or were patients with first-episode schizophrenia who were not previously treated with antipsychotics and had a Clinical Global Impression Scale score ≥4 [33]. All patients were admitted for inpatient treatment. Exclusion criteria were presence of any neurological, neurodegenerative, autoimmune, inflammatory disease (including acute infection or period of 3 months within the infection); cancer; diabetes mellitus; obesity; history of cerebrovascular event; history of head trauma with cognitive sequelae; epilepsy; intellectual disability; alcohol and drug dependence; existence or suspicion of organic basis for mental disorder (confirmed by previous complementary diagnostics or suspicion based on clinical evaluation); use of immunomodulatory and hormone drugs as well as pregnancy and lactation. Healthy individuals were recruited from the staff members of psychiatric hospitals involved in the research or community members who met the following criteria: age between 18 and 65 years. Exclusion criteria for healthy controls were the same as those for the patients. Besides that, they did not meet the criteria for any mental disorder according to ICD-10, did not have a history of disorder from a broader psychosis spectrum, did not have first- and second-degree relatives who have disorders from a broader psychosis spectrum and did not take any psychotropic medications or psychoactive drugs 4 weeks prior to the recruitment in the study. Schizophrenia patients and healthy controls were matched for age and sex. A total of 128 participants finished the study and were included in the analysis while 9 patients and 4 healthy individuals dropped out either because they were lost to follow-up for any reason or due to later confirmed or developed exclusion criteria (e.g., confirmation of the organic cause of illness or infection). There was a subgroup of 16 first-episode antipsychotic-naive schizophrenia patients out of 64 recruited schizophrenia patients that we segregated for the separate analysis. The baseline characteristics for all participants are presented in Table 1.

### 2.2. Study Design

This was a prospective, observational, case-control study. During the initial visit, all participants signed informed consent, completed screening and were subjected to the semi-structured psychiatric interview for the collection of the data relevant for the study Body mass, height, waist circumference and blood pressure were also measured. For schizophrenia patients, these data were collected both in the acute phase of illness and after the achievement of remission. All patients underwent clinical assessments using psychometric scales, and scores were recorded at two timepoints, at the moment of admission and at the moment of clinical remission. The following psychometric instruments were administered: Clinical Global Impression Scale (CGI), Positive and Negative Syndrome Scale (PANSS), Personal and Social Performance Scale (PSP), Global Assessment of Functioning Scale (GAF), Calgary Depression Scale for Schizophrenia (CDSS) and Brief Assessment of Cognition in Schizophrenia (BACS) for which data will be published elsewhere.

### 2.3. Blood Sampling

Blood sampling was performed for all participants under fasting conditions, between 7 and 9 am. Blood samples for the analysis of tryptophan, kynurenine and kynurenic acid were collected using lithium-heparin containing tubes. For the analysis of IL-1β, IL-8 and IFN-γ (for which the data will be published elsewhere), EDTA-containing tubes were used. These samples were transported using a cold chain to the laboratory (distance from clinical sites 50 and 750 m, respectively) where they were immediately centrifuged (15 min, 300× *g*). The supernatant plasma was removed and stored in microtubes at −80 °C until analysis. Blood sampling was performed twice for schizophrenia patients—in the acute phase after the admission and at the moment of clinical remission—while for healthy controls blood sampling was performed during the initial visit.

### 2.4. Tryptophan, Kynurenine and Kynurenic Acid Measurement

TRP, KYN and KYNA were measured in Li-Heparin plasma samples by the high-performance liquid chromatography (HPLC) method [34].

#### 2.4.1. Sample Preparation

The sample preparation procedure was carried out by mixing 150 μL plasma with an equal volume of 0.6 mol/L HClO_4_. Mixtures were vortexed and centrifuged at 12,000 rpm for 5 min at room temperature to principate and separate proteins. The supernatant was transferred into a vial specific for the autosampler of the HPLC system.

#### 2.4.2. Chromatographic Conditions

Analyses were performed on the HPLC system Agilent Technologies 1200 Series with a binary pump using two detectors: diode-array detector (DAD) and fluorescence detector (FLD). Separation of analytes was achieved using pre-column Eclipse XDB C18 (4.6 mm × 12.5 mm × 5 μm) and Zorbax Eclipse XDB C18 (4.6 mm × 150 mm × 5 μm) column. Detection was performed by DAD at 365 nm for kynurenine and by FLD at excitation 344 nm and emission at 398 nm for kynurenic acid and tryptophan.

The mobile phase was composed of 20 mmol/L Na acetate, 3 mmol/L Zn acetate and 7% acetonitrile, prepared daily and filtered through a 0.45 μm membrane filter and degassed by ultrasonic bath. The flow rate was 1 mL/min, and the volume of injection was 100 μL. Separation was achieved at room temperature. The total time for plasma sample analyses was 40 min. Elution profiles of the representative human plasma sample and standards are presented in Supplementary Figures S1 and S2, respectively. Peak identification was performed by comparing retention times of the eluting peaks with the known standards. The concentrations of analytes were calculated from peak areas. The linearity of the method was determined by seven-point calibration. Concentrations of standards were 0.5, 1, 2, 4, 6, 8 and 10 μmol/L for kynurenine; 5, 10, 20, 40, 60, 80 and 100 nmol/L for kynurenic acid; and 5, 10, 20, 40, 60, 80 and 100 μmol/L for tryptophan. Linearity curve, correlation coefficients, retention times and residual standard deviation values are presented in Supplementary Table S1.

The precision of measurements was examined by six replicate injections of the same standard solutions. The concentrations of standards were: 4 μmol/L for kynurenine, 40 nmol/L for kynurenic acid and 40 μmol/L for tryptophan. The coefficients of variation for retention time and peak areas were 0.092 and 0.665 for kynurenine, 0.058 and 1.16 for kynurenic acid and 0.047 and 0.984 for tryptophan. The accuracy of the method was determined by addition of known quantities of KYN, KYNA and TRP standards to plasma before deproteinization. Added concentrations for kynurenine were 2.5, 5 and 10 μmol/L; 25, 50 and 100 nmol/L for kynurenic acid; and 25, 50 and 100 μmol/L for tryptophan. The recovery for kynurenine was 90.46%, for kynurenic acid 96.72% and for tryptophan 92.31%. The limit of detection and quantification were determined mathematically, as three and ten times the standard deviation of noise over the time range of eluting peak. The limit of detection for kynurenine was 0.086 μmol/L and for kynurenic acid and tryptophan 0.025 nM. The limit of quantification for kynurenine was 0.288 μmol/L and for kynurenic acid and tryptophan 0.084 nmol/L. Chromatograms of kynurenine (KYN), kynurenic acid (KYNA) and tryptophan (TRP) in plasma and standards are presented in Supplementary Figures S1 and S2, respectively. Calibration curves for kynurenine (KYN), kynurenic acid (KYNA) and tryptophan (TRP) determination are provided in the supplementary material as Supplementary Figures S3–S5, respectively.

### 2.5. Statistical Analysis

Numerical data were presented as mean with standard deviation or with median with 25th and 75th percentile. Categorical variables were summarized by absolute numbers with percentages. The chi-square test was used to test differences in sociodemographic characteristics of patients with schizophrenia and the control group. Differences in clinical characteristics of patients with schizophrenia in the acute phase and at remission were analyzed using a paired *t*-test. Differences in concentrations of kynurenine pathway derivatives in the plasma of all and drug-naive schizophrenia patients between acute phase and remission were analyzed by the Wilcoxon paired test. Correlations between cytokines and levels of kynurenine pathway metabolites and clinical variables were evaluated using Spearman’s correlation analyses and are presented by correlation coefficients (rho). In all analyses, the significance level was set at 0.05. Statistical analysis was performed using IBM SPSS statistical software (SPSS for Windows, release 25.0, SPSS, Chicago, IL, USA).

## 3. Results

### 3.1. Sociodemographic and Baseline Characteristics

A total of 128 patients, of which 64 were diagnosed with schizophrenia (study group) and 64 were without any known mental health conditions (control group), were included in the study. Groups were matched according to age and gender. There were no significant differences among schizophrenia patients and controls in terms of age (36.72 ± 10.46 vs. 36.61 ± 10.51 years), gender distribution (57.8% males vs. 57.8% males), comorbidities, leukocytes number (6.93 ± 2.32 vs. 5.58 ± 1.52 × 10^9^/L) and BMI (24.58 ± 4.40 vs. 25.45 ± 4.28). Median of number of previous hospitalizations was 3 (1–5). Twenty-five percent of schizophrenia patients were drug naive; 31.3% were treated with clozapine (CLO) during the study. Sociodemographic and clinical characteristics of patients with schizophrenia in comparison to a control group are presented in Table 1.

### 3.2. Clinical Characterization of Schizophrenia Patients

There was a significant improvement in the clinical status of schizophrenia patients at remission (vs acute phase), as revealed by assessment of a PANSS positive scale, PANSS negative scale, PANSS general psychopathology scale, total PANSS score, CGI, GAF, CDSS and PSP scales (Table 2).

### 3.3. Levels of Tryptophan, Kynurenine and Kynurenic Acid

Differences in concentrations of tryptophan, kynurenine and kynurenic acid in the plasma of all and drug-naive schizophrenia patients between the acute phase and remission are presented in Table 3.

The levels of tryptophan, kynurenine and kynurenic acid in individuals diagnosed with schizophrenia were found to be significantly lower during the acute phase of the illness and remission when compared to levels observed in a control group (*p* < 0.001). There was no significant change in the levels of kynurenine and kynurenic acid between the acute phase and the remission (*p* > 0.05), while levels of tryptophan significantly decreased between the acute phase and the remission (*p* < 0.001). There was no significant difference in the plasma kynurenine/tryptophan ratio between all schizophrenia patients and healthy controls at any studied timepoint or between the acute phase and remission (*p* > 0.05).

There was no significant difference in the levels of tryptophan, kynurenine, kynurenic acid and the kynurenine/tryptophan ratio in the plasma of drug-naive and previously treated schizophrenia patients (*p* > 0.05). There was a significant difference in the plasma kynurenine/tryptophan ratio between the acute phase and remission in drug-naive schizophrenia patients (*p* > 0.05).

### 3.4. Plasma Levels of Kynurenine, Kynurenic Acid and Tryptophan in Subgroups of Schizophrenia Patients Treated and Non-Treated with Clozapine

Differences in concentrations of kynurenine pathway derivatives in the plasma in subgroups of schizophrenia patients, treated (CLO) and non-treated with clozapine (w/o CLO) in the acute phase and at remission are presented in Table 4. In the CLO and w/o CLO subgroup of patients, there was no difference in levels of KYNA and tryptophan neither in the acute phase nor in remission (*p* > 0.05). In the acute phase, there was no difference in levels of kynurenine between CLO and w/o CLO patients (*p* > 0.05), while in remission, the CLO subgroup had significantly lower levels of kynurenine in contrast to w/o CLO patients (*p* < 0.05) (Table 4).

### 3.5. Correlations among Plasma Concentrations of Kynurenine, Kynurenic Acid and Tryptophan in Sschizophrenia Patients and Clinical Parameters

Correlations among kynurenines and clinical status parameters according to PANSS in schizophrenia patients in the acute phase and at remission are presented in Table 5. In both the acute phase and remission of patients diagnosed with schizophrenia, levels of KYNA correlated negatively with a positive scale (r = −0.364 and r = −0.315, respectively), general psychopathology scale (r = −0.310 and r = −0.268, respectively) and total PANNS scale (r = −0.323 and r = −0.299, respectively) (Table 5).

Correlations of illness duration and number of hospitalizations with kynurenines in schizophrenia patients in the acute phase and at remission are presented in Table 6. There was a negative correlation of tryptophan levels with both the illness duration and number of hospitalizations in the acute phase of patients diagnosed with schizophrenia (r = −0.329 and r = −0.287, respectively). There were no correlations between illness duration and number of hospitalizations with levels of kynurenines in schizophrenia patients during remission (*p* > 0.05).

## 4. Discussion

Using a prospective, observational, case-control design, we explored the differences of tryptophan, kynurenine and kynurenic acid in the plasma of schizophrenia patients and healthy individuals in a study sample of matched and highly selected participants so that major confounders whose presence is known to affect the kynurenine pathway are excluded and controlled [15]. As a confounding factor, inflammation merits particular attention. Some research data indicate that kynurenine pathway metabolites are one of the missing links between inflammatory disturbances and schizophrenia pathophysiology and clinical symptomatology [10]. Proinflammatory cytokines like IFNγ, TNFα, IL1β, IL-2, IL-4, IL6, IL-13, TGFβ and IL12 are known peripheral and central activators of IDO, one of the two initial enzymes in the cascade of tryptophan degradation [35,36]. In the brain, inflammatory cytokines play an essential role in astrocyte activation, IDO activation and thus the production of kynurenic acid [37]. However, kynurenines are not exclusively subordinated to the effects of proinflammatory cytokines on astrocytes activation, but this relationship is rather bidirectional as there is evidence that some kynurenines have an anti-inflammatory role [38]. Both under physiological and pathological conditions, various proinflammatory cytokines pass the blood-brain barrier to differing extents [16]. This said, a complex relationship of kynurenines and inflammation markers between the periphery and central nervous system is ubiquitous both in healthy and schizophrenia individuals, but the extent of this interaction and the nature of disturbances is not yet fully understood. In individuals with schizophrenia, convincing evidence of elevated levels of proinflammatory cytokines, known to activate IDO, is noted both in the peripheral blood [39] and centrally [40]. Yet, the evidence that linking elevated concentrations of peripheral proinflammatory cytokines to disturbances in the kynurenine pathway is notably less persuasive [28,41]. In our study, we excluded subjects who had or developed any inflammatory condition like autoimmune, inflammatory disease or infection, which were also criteria in other studies exploring kynurenines in schizophrenia [19,31,42,43]. Significantly, a criterion that distinguishes it from other comparable studies is individuals with a recent history of infection within three months preceding the start of the study were also excluded. This decision was made considering that proinflammatory markers can remain active in the periphery even after recovery from infections [44]. Furthermore, unlike many studies [41], we excluded subjects with obesity and diabetes mellitus as these are known as states of low-grade chronic inflammation that lead to the increase of inflammatory markers [45,46]. A limitation of our study is we did not account for factors that are implicated to have a role both in the states of low-grade inflammation and direct involvement in the kynurenine pathway—physical exercise, gastroenteric microbiota and chronic stress [22,47].

As mentioned above (see Introduction), disturbances of peripheral levels of kynurenine metabolites are frequently reported, but data across the studies are very inconsistent, with recent metanalysis showing that reductions of most kynurenine pathway metabolites are observed in schizophrenia patients, with larger effects during acute/symptomatic illness and in older age [30,41], while two other meta-analysis showed that there is no difference between peripheral levels of tryptophan, kynurenine and kynurenic acid between schizophrenia patients and healthy individuals [28,41].

In our study, there was a strong significance for decreased plasma concentrations of kynurenine and kynurenic acid in schizophrenia patients, and these differences were not specific for illness state (acute/remission) and were present irrespective of schizophrenia episode and previous treatment (first-episode antipsychotic naive/previously treated) and with levels of kynurenine and kynurenic acid not correlating with illness duration and number of hospitalizations. Besides that, there was no difference in the KYN/TRP ratio between schizophrenia patients and healthy controls irrespective of schizophrenia episode and previous treatment (first episode antipsychotic naive/previously treated). Our results are in line with the study of Szymona et al. [42] who reported that KYNA levels were significantly lower in schizophrenia patients in comparison to healthy controls at the moment of admission and after four weeks of treatment and in remission of schizophrenia patients. Our study was in line with results from a longitudinal study that measured blood kynurenine pathway metabolites in patients with schizophrenia spectrum disorders and demonstrated that in the early stage of the disease TRP, KYN, KYNA and anthranilic acid levels were significantly reduced [43]. Our results were in line with other studies in terms of decreased kynurenic acid levels in schizophrenia patients, but kynurenine and tryptophan levels were either not measured or the result was not presented [48,49]. Results from our study were in accordance with a meta-analysis that showed downregulation of the kynurenine pathway in the peripheral blood of schizophrenia spectrum disorder patients, although it found that these differences were state and age specific (i.e., acute symptomatic states and in older age) which was not the case in our study [30]. A study conducted by Skorobogatov et al. [50] explored serum abnormalities of the kynurenine pathway in a large sample size of stable and acute schizophrenia and bipolar disorder patients, and it was demonstrated that schizophrenia patients had lower concentrations of tryptophan, kynurenine and kynurenic acid than healthy controls, which was in line with our results. However, in contrast to our study, the authors showed that concentrations of tryptophan, kynurenine and kynurenic acid as well as the KYN/TRP ratio were lower in acute than stabilized patients, although the limitation of the study was a comparison of two separate groups of acute and stabilized patients while we measured interindividual differences.

The breakdown of tryptophan to kynurenine reflects the level of activity within the kynurenine pathway [22], and studies have indicated that the increase in the kynurenine/tryptophan (K/T) ratio in the periphery is linked to central nervous system diseases and functions as a reliable biomarker of kynurenine pathway metabolism [51]. No difference in the KYN/TRP ratio between schizophrenia patients and healthy controls observed in our study is in contrast with results from studies that found higher plasma KYN/TRP ratios [52,53,54]. These discrepancies in results could be explained by either smaller study samples [52,53] or non-fasting conditions for blood collection since changes in blood levels of tryptophan are prone to variabilities in dietary intake [53,54].

As the biological specimen used for kynurenines detection and quantification in our study was plasma, a nuanced exploration of the differences between measuring blood kynurenines in serum and plasma warrants discussion in the light of new findings. A recent meta-analysis [41] demonstrated that kynurenine levels were higher in serum samples but lower in plasma samples of subjects with SCZ with no differences in kynurenic acid levels measured in plasma vs. serum [29]. Almulla et al. [55] in a comprehensive meta-analysis exploring kynurenines in schizophrenia revealed significant differences between concentrations of kynurenine and kynurenic acid between all three sources (CNS, serum and plasma). Differences in tryptophan concentrations were not significant between the three sources, while overall metanalysis showed decreased tryptophan concentrations in schizophrenia patients. One of the important findings from the study was that the KYN/TRP ratio was significantly increased in schizophrenia patients, indicating increased IDO activity with a large effect size in CNS, small effect size in serum and nonsignificant effect size in plasma, implicating that the plasma KYN/TRP ratio should not be used as an indicator of IDO activity. Also, significant differences in KYN/TRP ratios were observed between all three sources (brain, plasma and serum). The authors speculated that the reasons for the observed dissociation between plasma and serum levels of kynurenines could be different analytical end preanalytical factors such as interference of tube coating materials with tryptophan catabolites and that measuring tryptophan catabolites in plasma might not be the most accurate specimen for the evaluation of circulating kynurenines in the blood. A study published this year [56] was the first to use simultaneous measurement and direct comparison of tryptophan catabolites concentrations in serum and plasma in a cohort of healthy individuals. Reported results showed significantly lower levels of tryptophan, kynurenine and kynurenic acid in plasma than in serum, but the reported differences were ≤10%, indicating that sensitivity might be slightly better in serum but that both sources could be used. Since measurements in our study were performed only on plasma samples, we consider it a limitation and suggest that simultaneous measurements in both the serum and plasma of schizophrenia patients and healthy individuals would improve validity.

Our results showing decreased KYN and KYNA in schizophrenia patients irrespective of illness state, antipsychotic treatment, number of episodes or illness duration coupled with no differences in the KYN/TRP ratio could be interpreted as a signal that the kynurenine pathway is not dysregulated in the periphery. This interpretation is in line with conclusions from a recent tryptophan challenge study that showed that the effects of orally administered tryptophan on peripheral levels of kynurenine and kynurenic acid did not differ significantly between SCZ patients and HC and that both basal and stimulated levels of kynurenine and KYNA in the blood do not accurately mirror or serve as indicators for abnormal changes in tryptophan degradation in schizophrenia (SZ) [31]. Results from this study suggested that structural and/or functional abnormalities in the brain and not in the periphery contribute to central KP impairments associated with schizophrenia and suggested that these could be alterations in the blood-brain barrier [57] or irregular astrocyte function [58]. Our interpretation is also in line with the conclusions of a recent study that measured concentrations of tryptophan, kynurenine, kynurenic acid, 3-HK and quinolinic acid in both postmortem brain tissues and blood of schizophrenia patients and healthy individuals [39]. Our finding of dissociation between downregulated KYN and KYNA with no changes in KYN/TRP ratio should be corroborated in further studies on bigger samples and simultaneous measurements in plasma and serum but also considering other parameters of IDO activity like (KYN + KA)/TRP ratios.

No difference between concentrations of tryptophan, kynurenic acid and the KYN/TRP ratio was observed between patients treated and not treated with clozapine either in the acute phase or in remission while levels of kynurenine decreased in patients treated with clozapine. This was in line with results from Chiappelli et al. who did not find significant associations of clozapine treatment with either kynurenine or kynurenic acid levels in stable schizophrenia patients [19]. One study found that chronic risperidone treatment in rats did not have an impact on the change of brain kynurenic acid levels [26]. A recent study explored the effects of antipsychotic therapy on circulating kynurenines across the disease trajectory, and most of the differences established for kynurenine metabolites in the antipsychotic-naive patients became weaker after six months of antipsychotic treatment [43]. Our results suggest that disturbances of peripheral levels of tryptophan, kynurenic acid and kynurenine are unrelated to the type of antipsychotic treatment, but studies on bigger samples should corroborate this finding.

Data about correlation of the duration of illness (DOI) and peripheral tryptophan catabolites are sparse and inconsistent in the existing literature. Our study reported only a negative correlation between tryptophan levels and DOI, both in the acute phase and remission, while the concentration of kynurenine and kynurenic acid did not correlate with illness duration. Two studies that explored the correlation between the duration of illness and tryptophan catabolites had somewhat contradicting results. In the already mentioned large study conducted by Skorobogatov et al. [50], longer duration of illness contributed to an increase of kynurenine and a decrease of tryptophan both in the acute and stable phase, while kynurenic acid did not correlate with DOI. The other study showed only a negative correlation between plasma kynurenic acid levels and DOI [42]. Given the inconsistency in results and the lack of clarity in reporting the correlation between DOI and peripheral levels of kynurenines in schizophrenia, this factor needs to be explored further.

Kynurenic acid, with its pertaining effects on the glutamatergic and cholinergic system in the brain, has been implicated as the link between the kynurenine pathway disturbances and positive, negative and cognitive schizophrenia symptomatology [7,59,60]. However, it was demonstrated that the impact of kynurenic acid on the glutamatergic system cannot be attributed only to effects on glutamate or cholinergic receptors [61]. Kynurenic acid has notable effects on G protein-coupled receptor 35 (GPR35) expressed in the brain. A study showed that activation of GPR35 astrocytic receptors by kynurenic acid can reduce glutamate synaptic levels, thus impeding excitatory transmission and concluding that GPR35 modulation by KYNA significantly contributes to overall kynurenine acid effects on neuronal circuits functioning [62]. Furthermore, kynurenic acid activates aryl hydrocarbon receptors (AHR) [63]. Aryl hydrocarbon receptor has come into the focus of schizophrenia research because of some indices that dysregulation of AHR signaling could have potentially disruptive effects on GABA interneurons with expected effects on inhibitory brain signaling [61], as well as involvement in oxidative stress and neuroinflammation in astrocytes, a crucial brain compartment of kynurenic acid production [64]. Research investigating the correlation between levels of kynurenine pathway metabolites and schizophrenia symptomatology has not yet provided conclusive findings.

The negative correlation of peripheral levels of kynurenic acid and overall symptom severity and severity of positive and general psychopathology both in the acute phase and remission is a finding that is not replicated by other studies in totality, while one study found a negative correlation of kynurenic acid levels with severity of positive symptomatology [65]. In one study, the severity of psychotic symptoms was correlated with decreased levels of kynurenine and kynurenic acid [50]. One study found a negative correlation between kynurenic acid levels and the severity of negative schizophrenia symptomatology [66], while another study found no correlation between levels of kynurenine and kynurenic acid with psychiatric symptoms [19]. Our isolated finding of the negative correlation of peripheral levels of kynurenic acid and overall symptom severity and the severity of positive and general psychopathology is inconclusive, but it can be discussed, based on our previous notion, that these observed effects are rather mediated by factors that influence crossover of KP metabolites from the periphery to the central nervous system and central events that contribute to schizophrenia symptomatology.

Our study had certain limitations. First, although our study had a big enough sample, larger-scale studies would improve the validity of the findings. Our study had a subgroup of 16 first-episode antipsychotic-naive patients, so additional studies including a higher number of these subjects would add to the robustness of the results for this subpopulation. Furthermore, our study measured peripheral levels of metabolites only at two time points—acute phase and remission—so longitudinal studies are necessary for drawing more relevant conclusions. An important limitation of our study was that we used only plasma as the biological specimen for kynurenine pathway metabolites detection, and, in the light of the discussed dissociation between plasma and serum concentrations, we suggest that further studies should perform simultaneous measurements in serum and plasma to improve the validity of the results. Moreover, we did not collect data on physical exercise—a factor that is known to affect the kynurenine pathway [67,68]. Finally, our study has included only circulating levels of kynurenine pathway metabolites in the blood, which comprises only one segment of the complex interaction of tryptophan catabolism in the periphery. Further studies should also integrate in their design other compartments with kynurenine pathway activity in the periphery (e.g., peripheral organs) with special attention on new evidence for the involvement of the gut-microbiota-brain axis in central-peripheral crosstalk of the kynurenine pathway [22,69].

## 5. Conclusions

Key findings from our study are decreased KYN and KYNA concentrations in the plasma in schizophrenia patients irrespective of illness state, type of antipsychotic treatment, number of episodes or illness duration and no differences in the KYN/TRP ratio between schizophrenia patients and healthy individuals. These findings could be interpreted as indices that the kynurenine pathway might not be dysregulated in the periphery and that other factors contribute to observed disturbances in concentrations. However, as our study had certain limitations, we cannot draw definite conclusions. Further studies with simultaneous measurements of kynurenines in plasma and serum as well as other body compartments that participate in kynurenine pathway are needed.

## Figures and Tables

**Table 1:** Baseline characteristics of patients with schizophrenia in comparison to a control group.

Variable	Group	
	Control (*n* = 64)	Schizophrenia (*n* = 64)
Gender, *n* (%)		
Male	37 (57.8)	37 (57.8)
Female	27 (42.2)	27 (42.2)
Age, mean ± SD	36.61 ± 10.51	36.72 ± 10.46
Smoking status, *n* (%)		
Non-smoker/Ex-smoker	43 (67.2)	25 (39.1)
Smoker	21 (32.8)	39 (60,9)
Comorbidities, *n* (%)	16 (25.0)	10 (16.1)
Family history, *n* (%)		
Psychosis	/	36 (56.3)
Suicide	/	11 (17.2)
Illness duration (months), median (25th–75th percentile)	/	96 (37–204)
Drug naive, *n* (%)	/	16 (25.0)
Clozapine, *n* (%)	/	20 (31.3)
Number of hospitalizations, median (25th–75th percentile)	/	3 (1–5)
BMI, mean ± SD	25.45 ± 4.28	24.58 ± 4.40
Leukocytes (10^9^/L), mean ± SD	5.58 ± 1.52	6.93 ± 2.32

**Table 2:** Clinical characteristics of patients with schizophrenia in acute phase and at remission.

Variable	Score (Mean ± SD)	
	Acute	Remission
PANSS positive scale	32.17 ± 5.54	14.56 ± 4.01 *
PANSS negative scale	26.09 ± 6.75	16.66 ± 5.49 *
PANSS general psychopathology	60.34 ± 8.22	32.61 ± 7.03 *
Total PANSS score	118.45 ± 17.86	64.09 ± 13.80 *
CGI	5.73 ± 0.76	1.70 ± 0.46 *
GAF	24.95 ± 8.32	58.81 ± 8.91 *
CDSS	5.06 ± 4.46	2.47 ± 2.48 *
PSP	26.78 ± 9.04	62.64 ± 10.80 *

* *p* < 0.001 vs. acute.

**Table 3:** Concentrations of kynurenine, kynurenic acid and tryptophan in the plasma of all and drug-naive schizophrenia patients between acute phase and remission.

Metabolite	Acute Phase	Remission	Healthy Controls
Kynurenine (μmol/L)			
All SZ patients	1.52 (1.27–1.70) ^a^	1.40 (1.16–1.80) ^a^	1.90 (1.52–2.29)
Drug naive	1.45 (1.07–1.66) ^a^	1.42 (1.08–1.80) ^a^	
Kynurenic acid (nmol/L)			
All SZ patients	21.30 (16.22–28.83) ^a^	19.88 (14.60–26.58) ^a^	31.81 (27.12–41.32)
Drug naive	22.36 (19.89–31.11) ^a^	20.87 (15.45–29.31) ^a^	
Tryptophan (μmol/L)			
All SZ patients	40.77 (33.81–46.13) ^a^	33.32 (26.02–40.58) ^a,b^	46.88 (41.97–53.62)
Drug naive	43.21 (38.98–48.56)	33.81 (26.92–42.46) ^a^	
KYN/TRP ratio			
All SZ patients	0.04 (0.03–0.05)	0.04 (0.03–0.06)	0.04 (0.03–0.05)
Drug naive	0.03 (0.03–0.04)	0.04 (0.04–0.05) ^b^	

Results are presented as median values (25th and 75th percentile). ^a^ *p* < 0.05 vs. healthy controls. ^b^ *p* < 0.05 vs. acute phase.

**Table 4:** Differences in concentrations of kynurenine pathway derivatives in the plasma in subgroups of schizophrenia patients, treated (CLO) and non-treated with clozapine (w/o CLO), in acute phase and at remission.

Substance	Acute		Remission		Control
	W/o CLO	CLO	W/o CLO	CLO	
KYN (μmol/L)	1.54 ^a^ (1.31–1.75)	1.43 ^a^ (1.24–1.62)	1.44 (1.20–1.84)	1.18 * (1.10–1.42)	1.90 (1.52–2.29)
KYNA (nmol/L)	21.75 ^a^ (17.32–28.83)	19.15 ^a^ (15.31–29.24)	20.76 (15.22–26.63)	16.86 (12.04–26.08)	31.81 (27.12–41.32)
TRP (μmol/L)	41.71 ^a^ (34.38–46.13)	38.45 ^a^ (33.81–47.08)	35.86 (26.92–41.02)	29.54 (23.60–36.90)	46.88 (41.97–53.62)
KYN/TRP ratio	0.04 (0.04–0.06)	0.04 (0.03–0.06)	0.04 (0.04–0.06)	0.04 (0.03–0.06)	0.04 (0.03–0.05)

Results are presented as median values (25th and 75th percentile). * *p* < 0.05 vs. without CLO. ^a^ *p* < 0.05 vs. control.

**Table 5:** Correlations among clinical status according to PANSS and levels of kynurenines in schizophrenia patients in acute phase and at remission.

	PANSS								ΔPANSS
	Acute				Remission				
	Positive Scale	Negative Scale	General Psychopathology	Total Score	Positive Scale	Negative Scale	General Psychopathology	Total Score	
**Acute**									
KYN	−0.113	0.147	−0.022	0.005	−0.102	0.009	−0.011	−0.025	0.066
KYNA	−0.364 *	−0.189	−0.310 *	−0.323 *	−0.315 *	−0.222	−0.268 *	−0.299 *	−0.103
TRP	−0.162	−0.034	−0.048	−0.069	−0.216	−0.123	−0.221	−0.219	0.113
KYN/TRP ratio	0.132	0.162	0.092	0.129	0.177	0.095	0.182	0.189	0.001
**Remission**									
KYN					−0.806	−0.133	−0.180	−0.162	0.207
KYNA					−0.257 *	−0.076	−0.250 *	−0.204	0.029
TRP					−0.119	−0.067	−0.127	−0.118	0.119
KYN/TRP ratio					−0.007	−0.144	−0.091	−0.093	0.029

* Data are shown as correlation coefficients (rho) values; *p* < 0.05 (Spearman’s correlation).

**Table 6:** Correlations of illness duration and number of hospitalizations with kynurenines in schizophrenia patients in the acute phase and at remission.

	Illness Duration (Months)	Number of Hospitalizations
**Acute**		
Kynurenine	0.051	−0.125
KYNA	−0.095	−0.194
Tryptophan	−0.329 *	−0.287 *
KYN/TRP ratio	0.262	0.136
**Remission**		
Kynurenine	0.149	−0.021
KYNA levels	−0.038	−0.095
Tryptophan	0.003	−0.054
KYN/TRP ratio	0.145	0.048

* Data are shown as correlation coefficients (rho) values; *p* < 0.05 (Spearman’s correlation).

## Data Availability

The data that support the findings of this study are available on request from the corresponding author.

## Supplementary Materials

The following supporting information can be downloaded at: https://www.mdpi.com/article/10.3390/cells12242814/s1, Figure S1: Chromatogram of kynurenine (KYN), kynurenic acid (KYNA) and tryptophan (TRP) in human plasma; Figure S2: Chromatogram of kynurenine (KYN), kynurenic acid (KYNA) and tryptophan (TRP) standards; Table S1: Linearity curve, correlation coefficient, residual standard deviation (RSD) and retention time (RT) for HPLC determination of KYN, KYNA and TRP; Figure S3: Calibration curve for kynurenine (KYN) determination; Figure S4: Calibration curve for kynurenic acid (KYNA) determination; Figure S5: Calibration curve for tryptophan (TRP) determination.
