# Supplementary material for: Decreased Plasma Levels of Kynurenine and Kynurenic Acid in Previously Treated and First-Episode Antipsychotic-Naive Schizophrenia Patients
Source: Cells. 2023 Dec 11;12(24):2814. doi: 10.3390/cells12242814 (PMC10741951; doi:10.3390/cells12242814)

cells

MDPI

Supplementary Materials:

Figure S1: Chromatogram of kynurenine (KYN), kynurenic acid (KYNA) and tryptophan (TRP) in human plasma

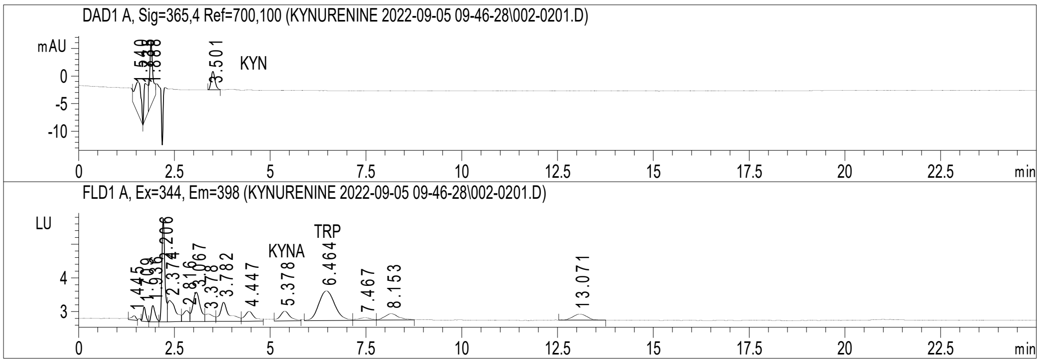

Figure S2: Chromatogram of kynurenine (KYN), kynurenic acid (KYNA) and tryptophan (TRP) standards

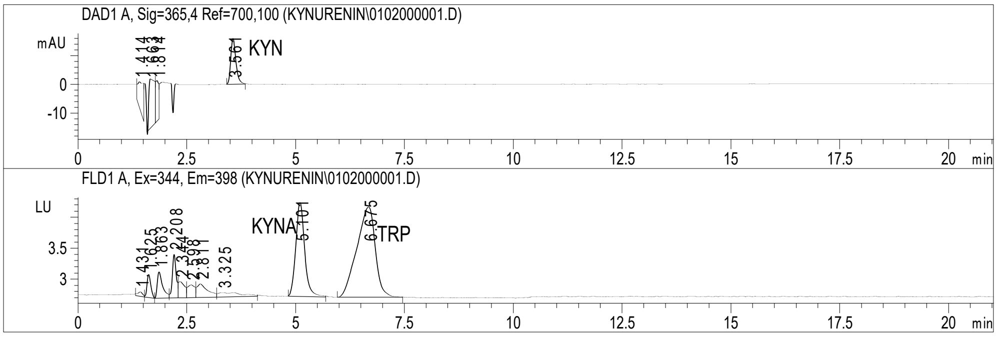

Table S1: Linearity curve, correlation coefficient, residual standard deviation (RSD) and retention time (RT) for HPLC determination of KYN, KYNA and TRP

Compound	Concentration range	Linearity curve*	Correlation coefficient	RSD	RT
KYN	0.5 -10 µmol/L	y= 25.23x	0.99998	0.99436	3.5
KYNA	5 -100 nmol/L	y=498.03x	0.99985	0.52174	5.3
TRP	5 -100 µmol/L	y=1.25x	0.99907	3.27371	6.5

*Linearty curve is given according to the equation y= mx. Linearity was determined by seven-point calibration.

Figure S3: Calibration curve for kynurenine (KYN) determination

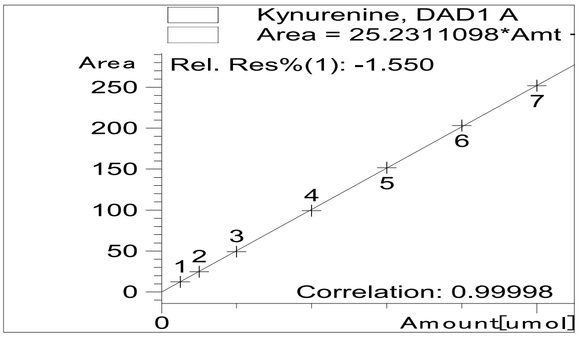

Figure S4: Calibration curve for kynurenic acid (KYNA) determination

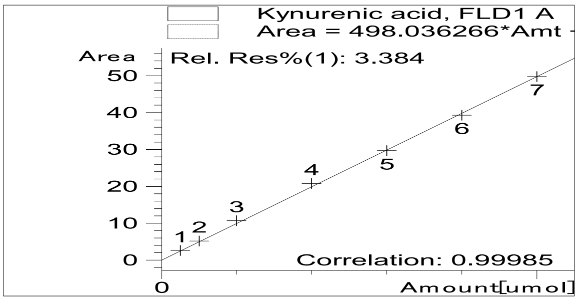

Figure S5: Calibration curve for tryptophan (TRP) determination

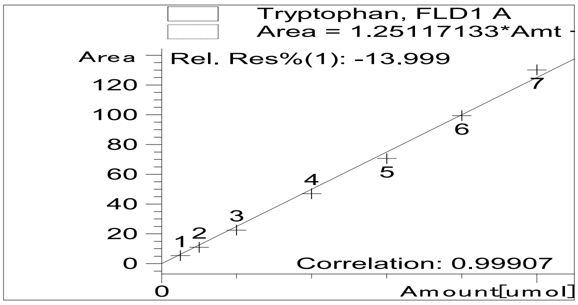

Supplement: Supplementary file 1 [file cells-12-02814-s001.zip › cells-2721867-supplementary.pdf]
